# Electrosprayed Core (Cellulose Acetate)–Shell (Polyvinylpyrrolidone) Nanoparticles for Smart Acetaminophen Delivery

**DOI:** 10.3390/pharmaceutics15092314

**Published:** 2023-09-13

**Authors:** Lin Xu, Hua He, Yutong Du, Shengwei Zhang, Deng-Guang Yu, Ping Liu

**Affiliations:** 1 School of Materials and Chemistry, University of Shanghai for Science and Technology, Shanghai 200093, China; 212203153@st.usst.edu.cn (L.X.); 213353175@st.usst.edu.cn (Y.D.); 2 The Third Affiliated Hospital, Naval Medical University, Shanghai 200433, China; 3 School of Health Science and Engineering, University of Shanghai for Science and Technology, Shanghai 200093, China; 222302325@st.usst.edu.cn; 4 The Base of Achievement Transformation, Shidong Hospital Affiliated to University of Shanghai for Science and Technology, Shanghai 200443, China

**Keywords:** coaxial electrospraying, cellulose acetate, core–shell nanoparticles, smart delivery, pulsatile release, sustained release

## Abstract

Smart drug delivery, through which the drug molecules are delivered according to the requests of human biological rhythms or by maximizing drug therapeutic effects, is highly desired in pharmaceutics. Many biomacromolecules have been exploited for this application in the past few decades, both in industry and laboratories. Biphasic release, with an intentional pulsatile release and a following extended release stage, represents a typical smart drug delivery approach, which aims to provide fast therapeutic action and a long time period of effective blood drug concentration to the patients. In this study, based on the use of a well-known biomacromolecule, i.e., cellulose acetate (CA), as the drug (acetaminophen, ATP)-based sustained release carrier, a modified coaxial electrospraying process was developed to fabricate a new kind of core–shell nanoparticle. The nanoparticles were able to furnish a pulsatile release of ATP due to the shell polyvinylpyrrolidone (PVP). The time cost for a release of 30% was 0.32 h, whereas the core–shell particles were able to provide a 30.84-h sustained release of the 90% loaded ATP. The scanning electron microscope and transmission electron microscope results verified in terms of their round surface morphologies and the obvious core–shell double-chamber structures. ATP presented in both the core and shell sections in an amorphous state owing to its fine compatibility with CA and PVP. The controlled release mechanisms of ATP were suggested. The disclosed biomacromolecule-based process–structure–performance relationship can shed light on how to develop new sorts of advanced nano drug delivery systems.

## 1. Introduction

Drug delivery is an applied scientific field that integrates knowledges and practices derived from multiple disciplines [1,2,3,4,5]. Its progress needs remarkable support from at least the following aspects: (1) novel active pharmaceutical ingredients (APIs) used to perform efficacious therapeutic actions or diagnosis [6,7,8,9], which act as the targeted delivery components; (2) safe and stable pharmaceutical excipients used as drug carriers, which help the API molecules to cross various obstacles to reach the lesion site [10,11,12,13,14,15]; (3) advanced techniques used to transfer the APIs and drug carriers into the designed medicated materials and the final dosage form for administration [16,17,18,19,20,21,22].

During the past few decades, cellulose and its derivatives have become popular as polymeric excipients used to develop a wide variety of drug delivery systems (DDSs, such as osmotic pump tablets, dispersible tablet, particles at all materials scales, films, and nanofibers) [23,24,25]. These successful outcomes mainly rely on the continuous introduction of advanced materials processing techniques into the pharmaceutic field to implement effective material conversion [26,27,28,29]. Those DDSs have been demonstrated to be able to provide all kinds of desired drug-controlled release profiles able to deliver loaded drug molecules in a designed manner, such as via targeted release at different levels (at the molecular level and organic level and in terms of drug-controlled release place), delayed release (in terms of the initial time point of drug-controlled release), fast release, sustained release and multiple-phase release (in term of drug-controlled release speed) [30,31,32,33,34].

**Figure 1:**
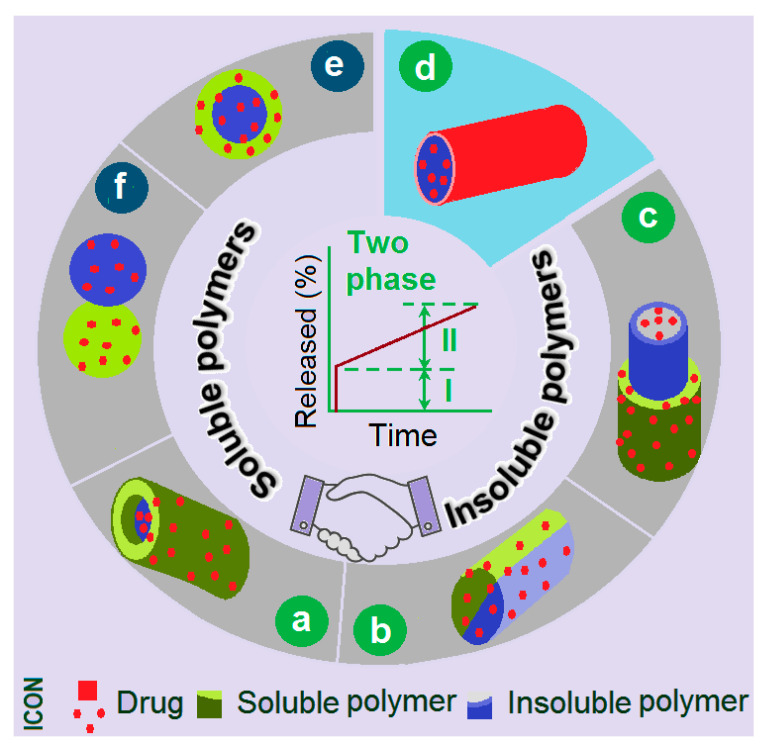
A diagram showing the biphasic release profiles provided by the combination of soluble and insoluble polymers in a certain structure: (**a**) core–shell nanofibers; (**b**) Janus nanofibers; (**c**) tri-layer core–shell nanofibers; (**d**) drug-coating nanofibers; (**e**) core–shell particles; (**f**) Janus particles.

Among the different kinds of drug-controlled release profiles, biphasic release, which is a fundamental multiple-phase release, is very useful for realizing the efficacious, safe, and convenient drug delivery goal. Most recently, a review by Zhou et al. discloses that biphasic drug-controlled release has the potential to experience a resurgence in popularity, and the supporting powers are the reasonable applications of complex micro- and nano-structures [35]. Figure 1 shows a diagram of the structures that can be relied on to provide drug biphasic release. Based on the fundamental design rule (i.e., the combined applications of soluble and insoluble polymeric excipients can be expected to provide the biphasic release), the soluble polymer can ensure a fast first-phase release (often through the erosion mechanism), and the insoluble polymer can endow a second phase of sustained release (often through a diffusion mechanism) [36,37,38,39,40,41,42]. However, determining the best way to organize the soluble and insoluble polymers in a suitable nanostructure poses a big challenge to the researchers.

One of the popular reasons for performing electrospinning is its ability to prepare core–shell [43,44,45,46,47], Janus [48,49], tri-layer core–shell [50], and tri-layer Janus nanofibers [51]; their combined structures; and fiber–particle hybrids in a single step and a straightforward manner [52,53,54,55,56]. Some of these electrospun structures are included in Figure 1a–d, and some of them have been explored with regard to providing biphasic drug release (e.g., fast release phase I and sustained release phase II), including core–shell nanofibers (Figure 1a), Janus nanofibers (Figure 1b), tri-layer core–shell nanofibers (Figure 1c), and drug-coating nanofibers (Figure 1d) [35]. Obviously, all of these biphasic release products are based on the electrospun nanofibers. Besides the standpoint of the inner structures of electrospun nanofibers that are utilized to furnish a biphasic release to smartly deliver a drug, they have been explored in terms of providing biphasic release through several other strategies. The first strategy is to organize different types of polymeric nanofibers in a layer-by-layer manner, with a fast release from the soluble polymer fibrous layer and a sustained release from the insoluble polymer fibrous layer. The second strategy is a combination of a electrospun soluble polymer layer to enable the fast release and a casting insoluble polymer layer to enable the second sustained release. The third strategy is the combination of electrospun soluble polymer nanofibers and the insoluble polymer particles, or vice visa [38]. In sharp contrast, biphasic release profiles derived from the electrosprayed particulate DDSs are very limited. As a sister technique of electrospinning, electrospraying has a similar capability as electrospinning, such as facile polymer-based material conversion, and a single-step and straightforward solidification [57,58,59,60,61]. Thus, we hypothesize that electrospraying can be explored to create double-chamber core–shell particles (Figure 1e) and Janus structures (Figure 1f) in a particulate format, via which method a drug is manipulated to be free from the particulate nanostructures in a double-phase manner to enable smart delivery.

During the development history of pharmaceutics, natural biomacromolecules have been very popular as drug carriers, and the reports about electrospun biomolecule-based DDSs are continuously improving [62,63]. Among all kinds of natural polymers, cellulose has made the most significant contributions to the development of novel DDSs. Particularly, some cellulose derivatives are soluble in water (such as HPMC series), which can be relied on to provide a fast release of the loaded drug molecules. In contrast, some cellulose derivatives are insoluble in water (such as ethylcellulose and CA), and they can be relied on for delayed release and sustained release profiles.

Based on the above-mentioned knowledge, in this study, a new type of electrosprayed core–shell microparticle, with CA used as the cellulose-based matrix in the core section, is developed to manipulate the biphasic release of acetaminophen (or paracetamol, ATP). A brand-new modified coaxial electrospraying process was developed for a continuous and energy-saving fabrication, which is important to the future large-scale preparations of electrohydrodynamic atomization (EHDA) products [64,65]. ATP was explored as a model drug. Its fast release in the first phase can result in a fast action in antipyretic and analgesic effects, and a second sustained release can provide a relatively constant blood ATP concentration to reduce the possible toxicity due to excessive dosage and oral administration times, thus increasing the patients’ compliance [66]. A series of physicochemical characterizations and in vitro and in vivo performance evaluations of the core–shell nanoparticles were carried out.

## 2. Materials and Methods

### 2.1. Materials

The raw ATP powders (white, purity of 99.8%) were purchased from the local Huashi Big Pharmacy (Shanghai, China). The polymeric matrices CA (Mw = 50,000) and polyvinylpyrrolidone (PVP) K10 (Mw = 8000) were obtained from Shanghai Haosheng Co., Ltd. (Shanghai, China). Methylene blue (as a color marker used to optimize the experimental parameters) and the organic solvents, including acetone, anhydrous ethanol, and dimethylacetamide (DMAc), were bought from the Shanghai First Reagent Factory (Shanghai, China). Water was doubly distilled immediately before use.

### 2.2. Methods

#### Modified Coaxial Electrospraying

In this study, the electrospraying apparatus and the key element, i.e., the spraying head, were homemade. Other elements in the apparatus included two syringe pumps (KDS100, Cole-Parmer, Vernon Hills, IL, USA), a power supply (ZGF2000, Wuhan Hua-Tian Co., Ltd., Wuhan, China), and a collector comprising a cardboard wrapped in an aluminum foil. The particulate collected distance was fixed at 20 cm, and the applied voltage was adjusted to maintain a continuous and robust fabrication process.

After some pre-experiments, three working fluids were prepared to conduct the electrospraying processes, and three particles were fabricated. Some experimental parameters used in these fabrications are listed in Table 1. The working processes were photographed using a digital camera (Canon PowerShot SX50HS, Tokyo, Japan).

### 2.3. Characterizations

#### 2.3.1. Morphology and Inner Structure

The morphologies of electrosprayed particles were assessed using a scanning electron microscope (FEI Quanta G450 FEG, Inc., Hillsboro, OR, USA). Before evaluation, the samples were evenly spread on a double-sided conductive tape and gold sputter-coated under nitrogen to render them electrically conductive. The pictures were taken at an excitation voltage of 10 keV. The inner structures of particles P3 were assessed using a transmission electron microscope (TEM, JEM2100F, JEOL, Tokyo, Japan). The samples were prepared by fixing a lacy carbon-coated copper grid to the collector for about 2 min.

#### 2.3.2. Physical State and Compatibility

A Bruker D8 ADVANCE diffractometer (Bruker, Bremen, Germany) was exploited to conduct the X-ray diffraction (XRD) analyses. The XRD patterns were achieved at the rotation range of 10 to 60°, with a step value of 0.02° and a rotation rate of 5°/min. The Attenuated Total Reflection-Flourier Transformation Infrared Spectroscopy (ATR-FTIR) was carried out using a Spectrum 100 spectrometer (Perkin-Elmer. Waltham, MA, USA). The scanning was implemented from 4000 to 500 cm^−1^, with a resolution of 2 cm^−1^ and a repeated time of 8.

### 2.4. Drug Encapsulation Efficiency and the In Vitro Drug Release Profiles

The encapsulation efficiency (EE%) was measured by extracting the ATP from the prepared particles as follows: accurately weighted particles of 1.0 g were dissolved into a 100-milliliter mixture of anhydrous ethanol and acetone at a volume ratio of 1:1; 1.0 mL of the solution was dripped into a 1000-milliliter phosphate buffered solution (PBS, pH 7.0, 0.1 M). After filtration using a 0.22-micrometer Millipore film, the solution was measured using a UV-vis Spectrophotometer (UV-2102PC, Unico Instrument Co. Ltd., Shanghai, China). The maximum absorbance of ATP was identified at 243 nm, which was exploited for its quantitative measurements. The encapsulated ATP in the electrosprayed products was calculated using the pre-determined calibration standard equation. The value of EE% could be achieved using the following Equation (1):

(1) EE(%)=WmWp×100%

where EE is the entrapment efficiency, W_m_ is ATP measured in the particles, and W_p_ is the ATP added in the working fluids. All measurements were performed in triplicate.

The in vitro dissolution tests were conducted according to the Chinese Pharmacopoeia (2020 Ed.). The paddle method was carried out using a RCZ-8A dissolution apparatus (Tianjin University Radio Factory, Tianjin, China) with seven vessels. The tests’ conditions included a rotation of 50 rpm and a dissolution medium temperature of 37 ± 1 °C. The dissolution media were 600 mL of PBS. At pre-determined time points, a 5.0-milliliter dissolution medium was withdrawn to perform sampling, and an equal fresh medium was added to maintain a constant volume. The absorbance levels of the samples were measured, and the released amount of ATP in the bulk solutions could be calculated using the calibration curve.

To disclose the molecular behaviors, a drop of water was dripped onto the collected particles P3 on the aluminum foil, which was later freeze-dried using a Biosafer-10A desktop freeze-drying machine (Shanghai, China). The residue core particles P3 were also freeze-dried for comparison.

## 3. Results and Discussion

### 3.1. The Modified Coaxial Electrospraying Process

**Figure 2:**
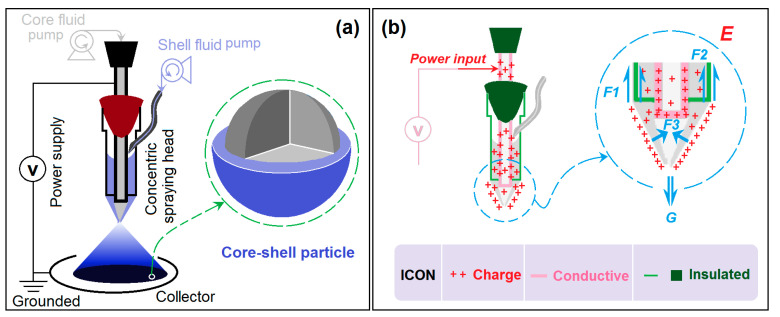
Modified coaxial electrospraying and its special spraying head: (**a**) a sketch exhibiting the modified coaxial electrospraying apparatus and process, which is characterized using a detachable concentric spinneret to generate core–shell particles; (**b**) the mechanism of the electrostatic energy transference and the formation of a Taylor cone at the tip of the spraying head.

Both electrospraying and electrospinning form part of the EHDA methods, where electrostatic energy is exploited as a fabrication tool to create solid products [67,68,69,70]. Thus, the spinneret or spraying head is the most important element in an electrospraying system due to the convergent places for all of the working fluids and electrostatic energy [71,72]. Compared to the traditional electrospinning and electrospraying methods, via which a full metal (often stainless steel) is exploited, the present work explored a specially designed spraying head. Other elements are similar, such as the syringe pumps, high voltage supply, and the plate collector composed of a cardboard wrapped in aluminum foil (Figure 2a).

In the detachable concentric spraying head, only a small section of the inner metal capillary was left to connect to the power supply for the objective of energy saving. Figure 2b shows a diagram of the mechanism used to transfer the electrostatic energy to the shell working fluid to ensure the formation of the compound Taylor cone, via which method the modified coaxial electrospraying process was initiated. The most fundamental reason for this outcome is that the high voltage electrons have a tendency to aggregate and distribute on the surface between the shell working fluid and the environment. Under a series of forces (such as the gravity G, the adhesion force F1 on the outer pipe wall, the capillary pipette force F2, the surface tension of the shell fluid F3, and the force from the electric fields E) and when the high voltage is high enough to provide an electric force E able to overcome the forces drawing the fluids back to their capillaries, the electrospraying process would be initiated to solidify the working fluids into solid particles.

**Figure 3:**
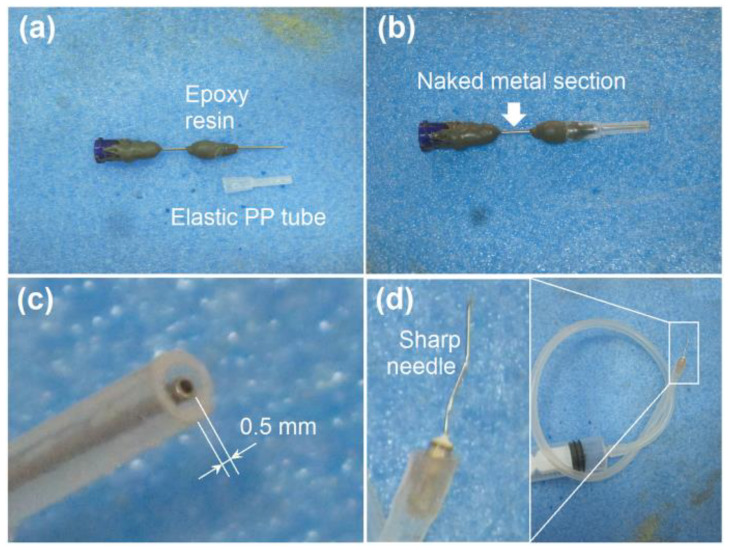
Organization of the detachable concentric spraying head: (**a**) a digital photo of the core tube and shell elastic PP tube; (**b**) the setting together of the core and shell tubes, with only a small section of the core metal capillary being naked to import the high electrostatic energy; (**c**) the inner stainless steel capillary slightly projected 0.5 mm out of the nozzle surface of shell PP tube; (**d**) a supplementary metal needle connected to an elastic silicon tube is exploited to send the shell working fluid into the spraying head.

The details of the organization of the detachable concentric spraying head are given in Figure 3. A digital photo of the core tube and shell elastic polypropylene (PP) tube is shown in Figure 3a. The core tube is a traditional mono-axial stainless steel capillary. In the elastic PP tube, one side is larger than the other side. Some epoxy resin (EP) was attached at the middle section of the inner metal capillary. When the two sections were set together, the optical image was found, as shown in Figure 3b, in which only a small section of the core metal capillary was not covered by EP or PP for the transference of the electrostatic energy from the power supply to the working fluids. In the nozzle of spraying head, the inner metal capillary was designed to project the outer PP tube by 0.5 mm to enable the easy encapsulation of the core fluid by the shell fluid (Figure 3c). Additionally, a supplementary metal needle (30 G, with inner and outer diameters of 0.16 mm and 0.31 mm, respectively) was required to connect the spraying head and the pump, which was utilized to convey the shell working fluid into the spraying head through an elastic silicon tube (Figure 3d).

**Figure 4:**
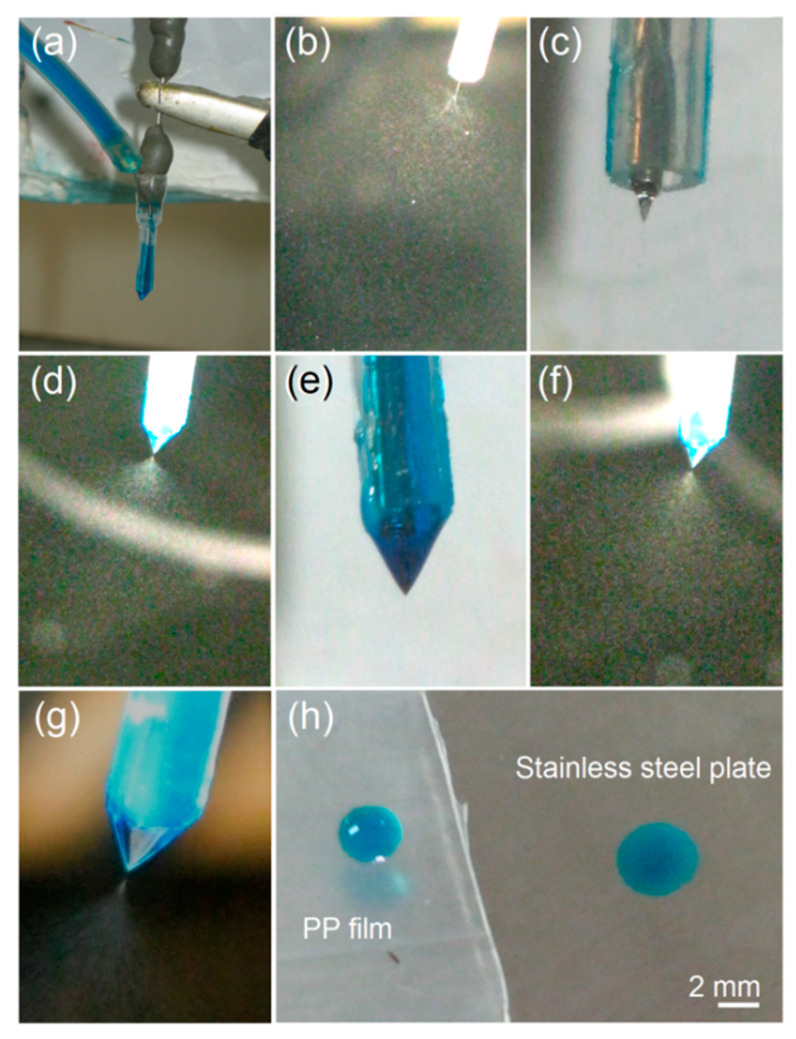
Digital pictures taken during the several electrospraying processes: (**a**) the connection between the concentric spraying head and the working fluids and electrostatic energy; (**b**,**c**) working processes used to prepare CA-PAT particles P1 and the Taylor cone, respectively; (**d**,**e**) working processes used to fabricate PVP-ATP particles P2 and the Taylor cone, respectively; (**f**,**g**) coaxial working processes used to fabricate core–shell hybrid particles P3 and the compound Taylor cone, respectively; (**h**) a simple experiment showing the interactions between the shell PVP-ATP working fluids and the PP film (**left**), as well as between the shell PVP-ATP working fluids and a stainless steel plate (**right**).

The typical observations of the fabrications of three kinds of electrosprayed particles are included in Figure 4. In Figure 4a, it is clear that the shell working fluid was directly sent to the spraying head through the syringe fixed on the pump. The shell working fluid, as indicated by the color marker of methylene blue (with a concentration of 0.001 mg/mL for pre-experiments), was guided to the spraying head using a high elastic silicon tube and a sharp needle. The electrostatic energy was transferred through an alligator clip.

When the shell working fluid was turned off, a typical single-fluid electrospraying process used to create the CA-ATP particles P1 can be observed, as shown in Figure 4b, in which the short straight fluid jet and the Coulomb explosion region are clear. In the photo under a magnification of 12×, the transparent small Taylor cone was also clear in Figure 4c. When the core fluid was switched off, a single-fluid electrospraying process used to create the PVP-ATP particles is exhibited, as shown in Figure 4d, in which the Taylor cone and Coulomb explosion can be discerned, whereas the straight fluid jet was almost entirely concentrated at one point. An enlarged Taylor cone of the electrosprayed shell fluid is shown in Figure 4e, and it is uniform in a blue color. The core–shell particles P3 were prepared under the pre-determined flow rates, and a typical electrospraying process was used in Figure 4f, which is similar to that used to create PVP-ATP particles P2 from the shell working fluid. However, the Taylor cone is completely different. Comparing Figure 4g,e indicates that the compound Taylor cone used in Figure 4g to fabricate the particles P3 contained two layers, with the inner layer having a slighter blue core, whereas the out layer had a deeper blue color than that of the homogeneous Taylor cone shown in Figure 4e.

The detachable concentric spraying head can benefit the coaxial electrospraying processes in several ways: (1) it is convenient for the arrangement and cleaning of the spraying head; (2) it is energy saving during the working process resulting from the insulated PP tubing, which has been previously demonstrated by conducting electrospinning [64]; (3) it is more facile in initiating the working process because the interfacial interaction between the shell working fluid and the PP tubing is smaller than that between the shell working fluid and the stainless steel capillary. A simple experiment was conducted to confirm this point. As shown in Figure 4h, a drop of the shell PVP-PAT working fluid is able to stand out on the PP film (with same texture as the shell PP tubing of the concentric spinneret), suggesting a very small interaction between the two elements. In contrast, when a drop of the shell working fluid was dripped on the surface of the stainless steel plate, the working fluid was spread all at once, indicating a fine compatibility and strong interfacial action between these elements.

### 3.2. The Morphologies and Inner Structures of the Electrosprayed Particles

**Figure 5:**
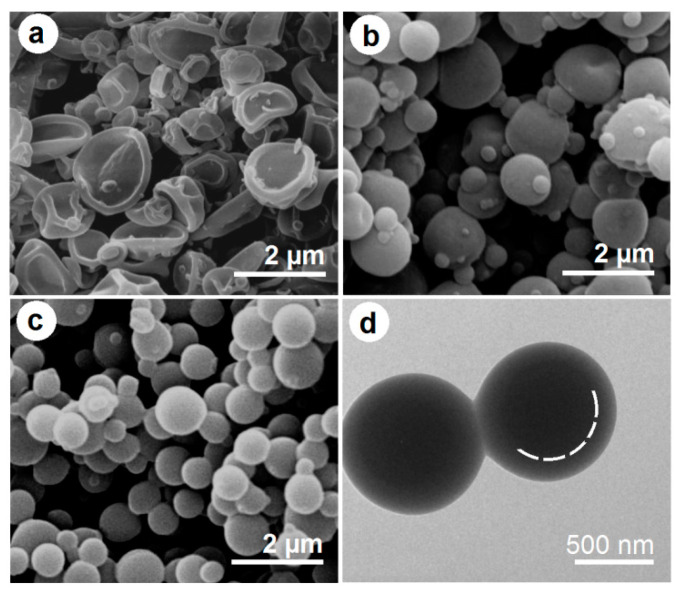
Evaluations of the electrosprayed particles: (**a**) SEM images of CA-ATP particles P1; (**b**) SEM images of PVP-ATP particles P2; (**c**,**d**) SEM and TEM images of the core–shell hybrid particles P3, respectively.

The morphologies and inner structures of the three types of electrosprayed particles are included in Figure 5. Figure 5a shows CA-ATP particles P1 derived from the single-fluid electrospraying of the core working fluid. Interestingly, these particles have an average diameter of 1.47 ± 0.51 μm and a sunken surface morphology. The formation of these recessed particles should have a close relationship with the film-forming property of CA, the easy evaporation of acetone and ethanol, and the continuous splitting of the sprayed working fluid. Within the Coulomb explosion region, the fast evaporation of the solvent from the surface of droplets would result in a semi-solid surface layer forming around the inner fluid. When the solid particles reach the collector, they essentially contain some solvent in their core section due to the fine film-forming capability of CA. Next, the entrapped solvent molecules would gradually reach the environment, and the barometric pressure would rapidly deform the initially round solid particles into a flat dented morphology. In contrast, the PVP-ATP particles derived from the single-fluid electrospraying of the shell working fluid show a solid and round morphology in Figure 5b. These particles have an average diameter of 1.21 ± 0.37 μm and have some satellites around them. Their surfaces are relatively smooth and continuous in nature.

The SEM image of the electrosprayed core–shell particles P3 is given in Figure 5c. These particles have a better quality in terms of the following two aspects: they have a round and solid morphology with a smooth surface and an average diameter of 0.87 ± 0.17 μm determined via estimation, and there are fewer satellites around the electrosprayed particles. The double-layer core–shell can be discerned via the TEM images in Figure 5d. One interesting phenomenon is that the core–shell particles P3 had a smaller diameter, a smaller diameter distribution, and a smoother surface than the particles prepared using the core and shell fluids in a single-fluid manner, although the total flow rates passing through the spraying head’s nozzle (1.0 mL/h) and the solutes within the working fluids were same (2% + 5% *w/v* in total). This result should be attributed to the fine compatibility between the shell fluid and the core fluid, the important role played by the shell working fluid in determining the Coulomb explosion, and its action as a useful transitional zone for the gradual exhaustion of the solvents in the core fluids.

### 3.3. The Physical State and Compatibility

**Figure 6:**
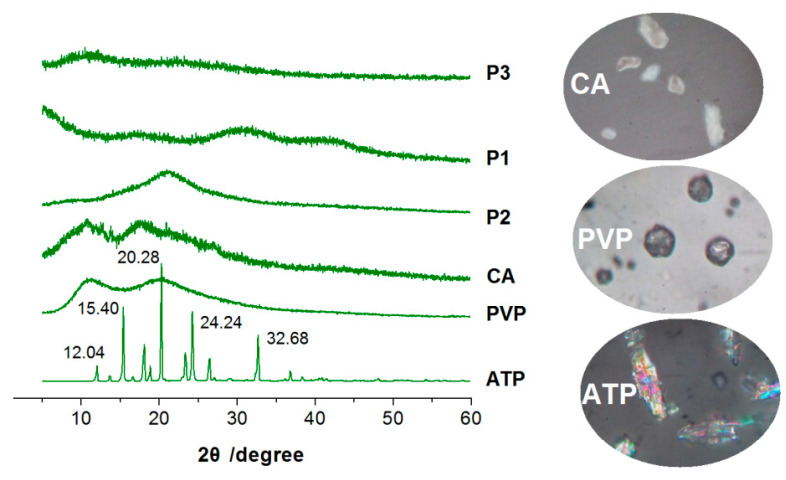
XRD patterns of the raw materials (APAP, PVP, and CA) and their electrosprayed products (P1, P2, and P3), with the three right-hand insets showing the images of the raw materials under a polarized optical microscope with a magnification of 200×.

One of the most difficult challenges in pharmaceutics is the dissolution and effective delivery of poorly water-soluble APIs [73,74]. The existence state of these poorly water-soluble APIs in the raw powders is mainly crystalline, which makes them difficult to disperse in the body fluids. Thus, an amorphous state is required for them to be dissolved and manipulated to be released in a controlled manner [75,76,77]. In this study, the raw drug ATP powders had a size smaller than 75 μm. Under the observation of a polarized optical microscope and a magnification of 200×, the particles showed a colorful image containing red, blue, green, and pink, as indicated in the bottom-right inset of Figure 6. In contrast, the raw powders of CA and PVP mainly exhibited different gray levels without the discerned color, suggesting they were amorphous polymeric excipients.

The XRD patterns of the three raw powders (CA, PVP, and ATP) are shown in Figure 6. The drug ATP may have sharp Bragg peaks (such as 15.40°, 20.28°, 24.24° and 32.68°) in its pattern, suggesting its crystalline state. The patterns of CA and PVP are mainly humps, indicating their amorphous state, which concurs with the observations made under a polarized optical microscope. Similarly, the XRD patterns of the electrosprayed particles P1, P2, and P3 only had blunt humps, without any discernable sharp peaks, hinting that they were amorphous composites or polymeric hybrids. The loaded ATP completely lost its original crystalline state after the process of electrospraying, regardless of the single-fluid process or the modified coaxial process used.

**Figure 7:**
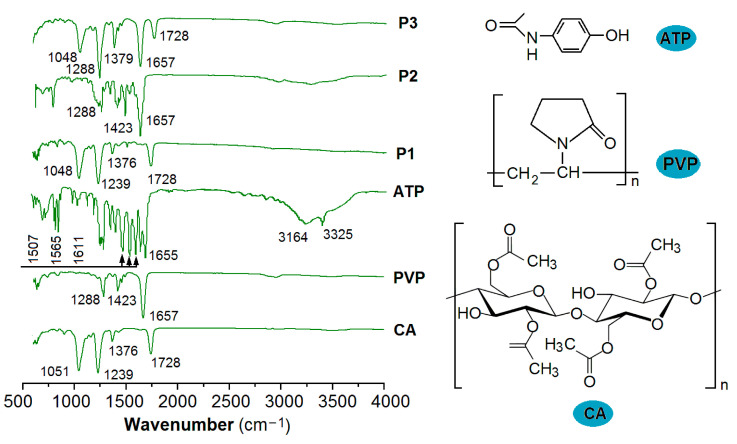
ATR-FTIR spectra of the raw materials (APAP, PVP, and CA) and their electrosprayed products (P1, P2, and P3), with the three right-hand insets showing the molecular formulas of the three starting components.

The amorphous state is a high-energy state over the crystalline state, and, thus, the long time period of stable existence needs fine compatibility between the co-existence components to prevent possible solid-phase separation [78,79,80]. FTIR spectra can be exploited to disclose the possible secondary interactions between the components, which include hydrogen bonding, electrostatic interactions, hydrophobic interactions, and Van Der Waals forces and are favorable to high stability [81,82,83]. The ATR-FTIR spectra of the raw materials (ATP, PVP, and CA) and their electrosprayed products (P1, P2, and P3) are included in Figure 7.

CA has its characteristic peaks at 1728, 1376, 1239, and 1051 cm^−1^. The drug ATP has the characteristic peaks of 3325, 3164, 1655, 1611, 1565, and 1507 cm^−1^. However, in their composite particles P1, the peaks of ATP have almost disappeared, leaving only those peaks of the host matrix of CA. These phenomena suggested that the secondary interactions have formed between CA and ATP molecules. As shown in the right-hand insets showing the molecular formulas of ATP and CA, both elements have hydroxyl groups (−OH) and carbonyl groups (−C=O). Thus, hydrogen bonding should be broadly presented between them. Meanwhile, the benzene ring of ATP and the long carbon chain of CA can have hydrophobic interactions. These favorable interactions would prevent the possible recrystallization of ATP molecules to maintain a molecular distribution state within the CA matrix for a long time period.

Similarly, in the spectra of electrosprayed PVP-ATP particles P2, all of the characteristic peaks of ATP have been greatly weakened or completely disappeared, leaving only the characteristic peaks of PVP, such as 1657, 1423, and 1288 cm^−1^. The reasons should be the secondary interactions between PVP molecules and ATP molecules. In the molecular formula of PVP, it is clear that there are many carbonyl groups (−C=O) through which hydrogen bonds can easily be formed by providing electron clouds to the protons in the hydroxyl groups (−OH) in ATP molecules.

In the spectra of the electrosprayed core–shell particles P3, as we anticipated, no obvious characteristic peaks of ATP can be discerned. The sharp peaks show a superposition of PVP (1657 and 1288 cm^−1^) and CA (1728, 1379, and 1048 cm^−1^), suggesting that the particles P3 are a combination of particles P2 and P1. In other words, the particles P3 are core–shell nanohybrids composed of two kinds of nanocomposites, i.e., the shell PVP-ATP composites and the core CA-ATP composites.

### 3.4. The Drug EE% and In Vitro Release Profile

The three kinds of electrosprayed monolithic particles P1, P2, and core–shell hybrid particles P3 have an EE% value of 100.4 ± 3.4%, 99.5 ± 2.3%, and 100.1 ± 3.1%, respectively. It was anticipated that all ATP molecules were successfully encapsulated into the polymeric matrices CA and PVP because that the EHDA process was essentially a physical drying process and the drying rate was extremely rapid [84]. During the fast materials conversion processes, the ATP molecules have little chance to escape from the working fluids and deposited particles due to no physical property of sublimation.

**Figure 8:**
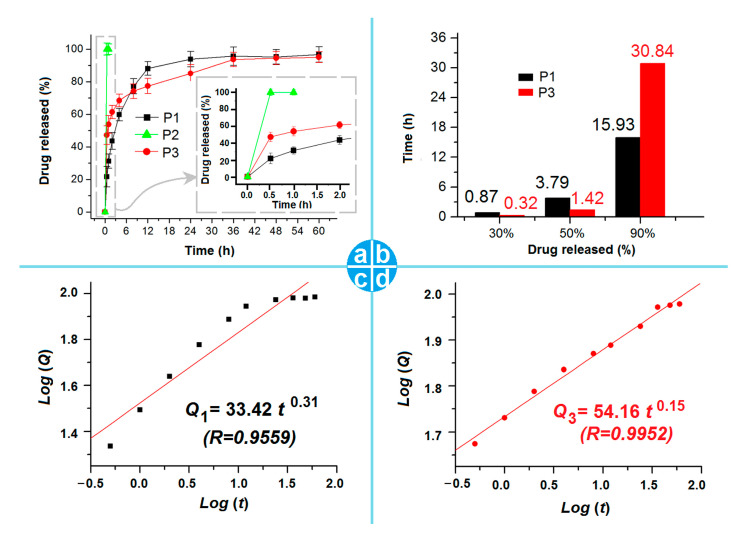
In vitro drug release profiles: (**a**) the full time scale release of accumulative release percentage vs. time points, with the inset showing the release profiles of the first 2 h; (**b**) the times needed to achieve a certain percentage release (30%, 50%, and 90%); (**c**,**d**) the drug release mechanisms for microparticles P1 and P3, respectively.

The ATP release profiles from the electrosprayed particles P1, P2, and P3 are included in Figure 8a. Apparently, the PVP-ATP particles P2 exhibited a typical pulsatile release, whereas the CA-ATP particles and the core–shell particles P3 showed a sustained release over a time period of 60 h. In the inset of Figure 8a, the release contents in the first half an hour were 21.7 ± 6.3%, 100.4 ± 3.7%, and 47.2 ± 5.7% for electrosprayed particles P1, P2, and P3, respectively. In Figure 8a, the drug release curves for the particles P1 and P3 are similar, likely due to the same trends. Thus, based on the in vitro release profiles, the interpolation method was exploited to disclose the time needed to release a certain percentage of the loaded ATP (30%, 50%, and 90%). The interpolation results are included in Figure 8b. The times needed for the samples of particles P1 and P3 to release a content of 30% loaded ATP were 0.87 h and 0.32 h, respectively, suggesting that the core–shell particles P3 were able to furnish a faster release of ATP than the composite CA-ATP particles P1. The times needed for P1 and P3 to release 50% of the loaded ATP were 3.79 h and 1.42 h, respectively, further suggesting the fast release of ATP from the core–shell particles P3. However, the times needed for particles P1 and P3 to release 90% of the loaded ATP were 15.93 h and 30.84 h, respectively. This finding means that about 40% of the loaded ATP in particles P3 were released in a sustained manner over a time period of over 29 h. This smart delivery method is favorable in terms of keeping a long time period of a constant blood drug concentration, as well as the compliance of the patients, due to shorter drug administration times.

The polymeric carriers’ properties are often relied on to manipulate the drug release profiles [85,86,87]. The fast release of ATP from the electrosprayed PVP-ATP particles P1, as well as the first-phase pulsatile release of the core–shell particles P3, on one hand, should be an expected result of the easy dissolution of PVP. On the other hand, the small diameter of particles P2, the thin layer thickness of particles P3, and the amorphous state of ATP presented in the PVP matrix co-acted to promote the fast dissolution of ATP from the PVP matrix through an erosion mechanism.

CA is an insoluble polymeric excipient, which is frequently exploited to provide the drug-based sustained release profiles through various strategies (such as blended matrix, compressed tablets, coating films, osmotic pumps, and many CA-based nanoparticles, microparticles and nanofibers). Peppas’ equation [88] was utilized to regress the drug release data. The results of electrosprayed particles P2 and P3 are shown in Figure 8c,d, respectively. The equation for CA-ATP composite particles P1 is Q_1_ = 33.42 t ^0.31^, with a correlation coefficient of 0.9559 (Figure 8c). The respective equation for the electrosprayed core–shell hybrid particles P3 is Q_3_ = 33.42 t ^0.15^, with a correlation coefficient of 0.9952 (Figure 8d). Based on the values of exponents (0.31 and 0.15 for particles P1 and P3, respectively), the ATP released from the composite particles P1 and the core sections of particles P3 is controlled via the typical Fickian diffusion mechanism due to it having a value smaller than the critical value of 0.45.

### 3.5. The Biphasic Release Mechanism from the CA-Based Hydrogel Particles

**Figure 9:**
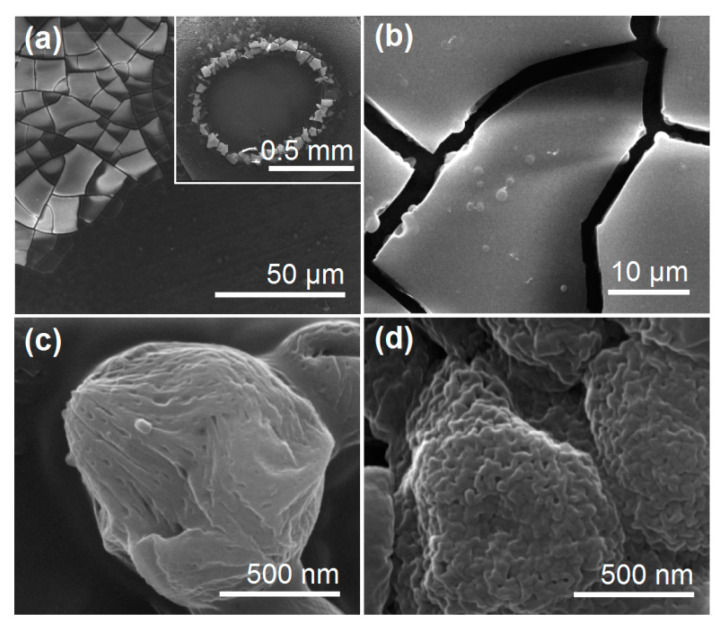
SEM observations regarding the behaviors of CA molecules during the drug-based sustained release processes: (**a**) the images of the residue film, which were formed by dripping a drop of water onto the collected electrosprayed particles P3 and drying them in a vacuum oven, with the upper-right-hand inset showing an image of the whole residues; (**b**) an enlarged SEM image of the residues; (**c**) a SEM image of a typical core section in the residues; (**d**) a SEM image of the core particles after the end of in vitro dissolution tests, which were sampled via a centrifuge and freeze-dried.

Although the ATP release from the CA matrices is manipulated via the Fickian mechanism, this is still an apparent result that mainly reflects the behaviors of ATP molecules during the drug release experimental process. The behaviors of those biomacromolecules during the drug-based sustained release have been seldom reported in the literature, which should be a very useful approach to design novel functional medicated nanomaterials. Figure 9a shows the images formed by dripping a droplet of water onto the collected electrosprayed particles P3 on the aluminum foil and performing vacuum drying. The upper-right-hand inset of Figure 9a shows a full reside image left by the water. Figure 9b shows an enlarged image, in which the solid PVP film membrane, due to its easy dissolution in water, cracks as a result of freeze-drying, and the buried core particles on the film surface and in the cracks are all clear.

The Images shown in Figure 9c show the surface morphologies of the core sections in the electrosprayed core–shell particles P3. There are special linear patterns and small pits on the solid surface. In contrast, the electrosprayed CA-ATP particles P1 have a very smooth and compact surface, as indicated via the SEM images in Figure 5a, due to the fine film-forming property of CA, although they have a flat and dented shapes. These observations suggested that the CA molecules organized in a looser state in the core sections of electrosprayed particles P3 than the case in electrosprayed CA-ATP particles P1. The SEM images of the core particles after the finish of the in vitro dissolution tests were also taken for comparison, and they are exhibited in Figure 9d. Apparently, there are more small openings and a bumpier surface morphology, hinting that the CA molecules had their own special behaviors during the ATP dissolution processes.

**Figure 10:**
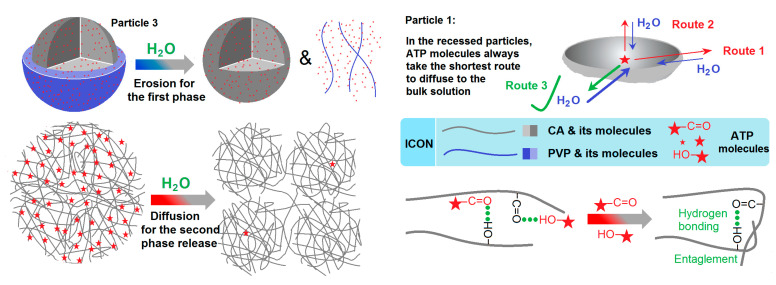
Molecular behaviors for disclosing the drug-based sustained release mechanism.

A sketch showing the molecule behaviors of both CA and ATP from the monolithic dented CA-ATP particles P1 and the core–shell hybrid particles P3 is depicted in Figure 10. For particles P1, the only matrix is CA, which is insoluble in water and hydrophobic in nature. When they are placed into the dissolution medium, the water molecules would penetrate the particles from all sides, via which method the insoluble CA gels were formed, and the particles would swell to a certain extent. Within the swollen insoluble gels, the ATP molecules would be free from the linkages with CA molecules and diffuse from the swollen particles to the outer dissolution medium due to the concentration differences. For an ATP molecule, theoretically, there are many routes that it can take to diffuse to the dissolution medium (for example, Route 1, Route 2, and Route 3 in Figure 10). However, it would always take the shortest route to more quickly reach the water molecules, as well as the easiest way to escape to the bulk solution.

For the core–shell particles P3, they would firstly remove the shell PVP-ATP sections through an erosion mechanism, and the ATP molecules would rapidly dissolve into the dissolution media along with the PVP molecules. Next, the ATP molecules encapsulated in the core CA matrix would gradually diffuse to the bulk solutions after the penetration of water molecules and the forming of insoluble gels of CA, which is similar to the behaviors of particles P1. However, there are four aspects that would exert influence on the ATP molecules’ behaviors. The first aspect is that the surface morphologies, as the surfaces of particles P1 are more compact than the core CA section of particles P3 for the above-mentioned reasons and, thus, more difficult for water to penetrate and gels to form. The second aspect is the fact that the core CA sections of particles P3 are more regularly round in shape, meaning that water molecule penetration and ATP molecule diffusion can easily be manipulated and predicted than the irregular shapes of particles P1. Thirdly, the core CA section of particles P3 should have a higher density than the particles P1, which can be discerned based on their diameters and the preparation of working fluid flow rates. A higher density means slower penetration rate of water and gel forming, as well as better sustained release of ATP molecules from the core CA matrices to the bulk solution. The last but nonetheless important aspect is the fact that the fast dissolution of the shell ATP molecules in the bulk solutions would greatly reduce the drug concentration gradients of the drug molecules free from the core CA-based gels to diffuse from the gels to the bulk solutions, thus leading to the elongation of the time period required for ATP molecule release in the second phase. The above four aspects regarding ATP, water, and CA molecular behaviors co-act to endow the core section of electrosprayed core–shell particles P3 with a better sustained release profile than those of the electrosprayed monolithic CA-ATP particles P1. Meanwhile, when the ATP molecules linked to the CA were cut off, the hydrogen bonds between the CA molecules would be formed based on the principle of proximity during the freeze-drying process, as depicted in the bottom-right-hand inset of Figure 10. This issue is the reason that the rough and uneven surfaces are shown in Figure 9d. The core–shell structure represents an inner–outer spatial relationship, which is frequently explored to develop new functional electrospun nanofibers [89,90,91,92]; thus, there are no doubts that electrosprayed core–shell structures would similarly enrich the novel functional structural nano- or micro-materials [93]. Furthermore, other advanced and traditional pharmaceutical techniques, such as Supercritical Fluid Technology [94,95,96] and tableting [97], can be combined with the advanced EHDA methods to develop ever-more novel structural dosage forms.

## 4. Conclusions

In this study, a homemade spraying head composed of both a polymer shell tube to enable energy saving and a metal core tube to enable easy fixing was designed to conduct a modified coaxial electrospraying process. Three kinds of electrosprayed particles were successfully fabricated using the same electrospraying apparatus: the monolithic insoluble particles P1 composed of a biomacromolecule CA and the drug ATP derived from a single-fluid electrospraying of the core solution; the monolithic soluble particles P2 comprising a soluble polymer PVP and the drug ATP from a single-fluid electrospraying of the shell solution; the core–shell hybrid particles P3 derived from the double-fluid coaxial electrospraying process. SEM and TEM images indicated that the shell working fluid could round up the CA-ATP core sections and ensure a loose surface to enable easy insoluble gel forming during the dissolution process. XRD patterns and ATR-FTIR spectra demonstrated that ATP had fine compatibility with both CA and PVP and was presented in all the three types of particles in an amorphous state. In vitro dissolution tests verified that the core–shell nanoparticles P3 were able to, on one hand, furnish a pulsatile release of ATP due to the PVP shell (the time cost for a release of 30% was 0.32 h). On the other hand, the core–shell particles P3 were able to provide a 30.84-h sustained release of the 90% loaded ATP, holding the promise of creating a smart drug delivery effect with a fast therapeutic action, a long time period of constant blood drug concentration, and, thus, shorter oral administration times that improve the compliance of the patients. Besides the ATP release mechanism from the CA matrices being identified as the typical Fickian diffusion mechanism, the gels’ forming behaviors, the water molecules’ behaviors, and the ATP molecules’ behaviors during the dissolution processes are suggested in detail. The protocols reported here paved the way for the design and fabrication of novel kinds of biomacromolecule-based drug delivery systems with distinct process–structure–performance relationships.

## Figures and Tables

**Table 1:** Experimental parameters used in the fabrication of electrosprayed particles.

No.	Electro- Spraying	Applied Voltage (kV)	Fluid Flow Rate (mL/h)		Theoretical Drug Content (%)	Products
			Core ^1^	Shell ^2^		
P1	Single-fluid	23	1.0	--	28.6%	Monolithic particles
P2	Single-fluid	16	--	1.0	28.6%	Monolithic particles
P3	Coaxial	18	0.6	0.4	28.6%	Core–shell nanoparticles

^1^ The core fluid consisted of 2.0 g of ATP and 5.0 g of CA in a 100-milliliter solvent mixture of anhydrous ethanol, acetone, and DMAc at a volume ratio of 1:4:1. ^2^ The shell solution was composed of 2.0 g of ATP and 5.0 g of PVP K10 in a 100-milliliter solvent mixture of anhydrous ethanol and acetone at a volume ratio of 6:4.

## Data Availability

The data supporting the findings of this manuscript are available from the corresponding authors upon reasonable request.
